# Exploring the Correlation Between Sleep Chronotype and the Volumes of Subcortical Structures and Hippocampal Subfields in Young Healthy Population

**DOI:** 10.3390/brainsci15030295

**Published:** 2025-03-11

**Authors:** Fahad H. Alhazmi

**Affiliations:** Department of Diagnostic Radiology, College of Applied Medical Sciences, Taibah University, P.O. Box 344, Almadinah Almunawarah 41477, Saudi Arabia; fhdhazmi@taibahu.edu.sa

**Keywords:** sleep chronotype, brain volume, subcortical structures, hippocampal subfields, hemispheric asymmetries

## Abstract

Background/Objectives: Chronotypes significantly influence sleep quality, daily performance, and overall activity levels. Although there is growing evidence indicating that individuals with a late chronotype are more likely to experience cognitive decline, the specific neural mechanisms that contribute to this risk remain unclear. This study aims to explore the relationship between morning and evening preferences and the volumes of subcortical structures in a young, healthy population. Methods: A total of 123 participants (80 females), aged between 18 and 35 years, were recruited. They underwent MRI scans and completed several self-reported assessments, including the morningness–eveningness scale of the Chronotype Questionnaire (ChQ-ME), the amplitude scale of the Chronotype Questionnaire (ChQ-AM), the Epworth Sleepiness Scale (ESS), and the Pittsburgh Sleep Quality Index (PSQI). Participants were classified into early chronotype (EC) and late chronotype (LC) groups based on their ChQ-ME scores. High-resolution T1-weighted imaging was utilized to analyze the volumes of subcortical structures and hippocampal subfields. Results: The volumetric analysis indicated that the LC group showed significant reductions in the right Caudate (*p* = 0.03) and the left SR-SL-SM (*p* = 0.03) compared to the EC group. Additionally, a notable leftward hemispheric laterality of the Subiculum (*p* = 0.048) was observed in the EC group relative to the LC group. Furthermore, the ChQ-AM revealed significant positive (r = 0.23) and negative (r = −0.19) correlations with the volumes of the left thalamus and right amygdala, respectively. The PSQI demonstrated a significant negative correlation (r = −0.21) with the right SR-SL-SM, while the ESS indicated a significant positive correlation (r = 0.24) with the left SR-SL-SM. Multiple regression analysis indicated that variations in daytime sleepiness are linked to the change of the left SR-SL-SM volume. Conclusions: Overall, the findings suggest that chronotype preferences are associated with the changes in the volumes of subcortical structures and hippocampal subfields and highlight the role of chronotypes in the neural mechanisms of these brain structures.

## 1. Introduction

A person’s chronotype refers to their natural inclination to sleep at specific times within a 24 h cycle. This preference is shaped by genetics [1] and influenced by the body’s circadian rhythm [2], dictating periods of wakefulness and rest [3]. Chronotypes play a significant role in determining sleep quality, daily performance, and overall activity levels [4]. Embracing one’s innate chronotype can enhance sleep quality, boost energy levels, and improve mood.

Chronotypes are generally categorized into three types: morning, evening, and intermediate. This classification can be determined through self-assessment tools like the morningness–eveningness scale of the Chronotype Questionnaire (ChQ-ME) [5] and amplitude scale of the Chronotype Questionnaire (ChQ-AM) [6]. Daytime sleepiness and sleep quality can be evaluated using the Epworth Sleepiness Scale (ESS) [7] and the Pittsburgh Sleep Quality Index (PSQI) [8], respectively. These self-report questionnaires have been extensively utilized to examine the impact of chronotypes on conditions such as obstructive sleep apnea [9], depression [10], bipolar disorder [11], and Parkinson’s disease [12].

Neuroplasticity, also known as brain plasticity, refers to the nervous system’s capacity to adapt its activity in response to internal or external stimuli by reorganizing its structure, functions, or connections [13]. This adaptability allows the brain to adjust to new experiences, learn new information, and recover from injuries [14]. Factors that influence brain plasticity include age [15], environment [16], and cognitive activities [17]. Research suggests a connection between brain plasticity and chronotype. Animal studies have demonstrated that circadian chronotypes influence hippocampal plasticity [18,19]. In humans, various studies indicate that the chronotype serves as a predictor for grey matter volume [20,21,22,23], white matter integrity [24,25,26], and brain connectivity [27,28,29].

For instance, Rosenberg et al. (2018) identified that differences in chronotypes correlate with specific neural substrates related to cortical thickness, surface areas, and folding patterns [20]. Additionally, Vulser et al. (2023) reported that eveningness is linked to changes in grey matter volume in the medial prefrontal cortex [21]. Zareba et al. (2022) found that circadian preference is associated with distinct structural cortical features [22]. Furthermore, Lapidaire et al. (2021) highlighted that a later wake-up time on weekends negatively affects adolescents’ emotional regulation abilities [23].

Regarding structural connectivity, it was found that circadian factors may play a more prominent role in WM integrity in emerging mood disorders [24]. Furthermore, Lee et al. (2022) reported that shift workers exhibited higher white matter integrity than did non-shift workers in the bilateral anterior cingulum [26].

In terms of brain functional connectivity, Mason et al. (2023) found that individuals with extreme early and late chronotypes exhibit distinct functional connectivity patterns [27]. Additionally, Wang et al. (2023) explored the relationships among chronotype, daily physical activity, and the functional connectivity between the default mode and ventral attention networks, indicating that this connectivity acts as a mediator between the chronotype and daily physical activity [28]. Furthermore, the functional connectivity within the default mode network mediates the relationship between the chronotype and sleep quality [29].

Subcortical structures of the brain are a varied collection of neural formations situated in the basal region, which are essential for a range of functions, including motor control, emotional processing, and cognitive activities [30]. The caudal hypothalamus and brainstem are parts of subcortical structures that are involved in controlling the alternation of sleep and wakefulness [31]. The link between the hippocampus and episodic memory is well recognized [32,33]; however, the functions of specific structures within the hippocampus related to particular tasks are still being developed [34]. They play roles in retrieving, encoding, and processing memory [35]. Hippocampal subfield atrophy has been reported in early psychosis [36] and Alzheimer’s Disease [37], as well as in chronic insomnia [38].

Despite the fact that increasing evidence suggests late-chronotype individuals are at increased risk of developing cognitive decline [39,40,41,42], the underlying neural mechanisms that confer risk are not well understood. Previous studies have focused mainly on unbiased whole brain voxel-based analysis that has several limitations, such as the fact that all MR images need to be acquired on the same MRI scanner, the difficulty of spatial normalizing atypical brains, and the less precise image registration of some brain tissue classifications [43]. Here, surface-based morphometry and region of interests (ROIs) analyses are used to assess the association between sleep chronotype and the volumes of subcortical structures and hippocampal subfields in a young healthy population. Here, it is hypothesized that a later chronotype would be associated with changes of subcortical structures and hippocampal subfields volumes.

## 2. Materials and Methods

### 2.1. Subject Selection and Scanning

In the current study, the data were sourced from the OpenNeuro database (https://openneuro.org/datasets/ds003826/versions/3.0.0, accessed on 20 November 2024) and they were collected in the course of two functional MRI (fMRI) projects (National Science Centre, Poland grants: Symfonia 2013/08/W/NZ3/00700 and Harmonia 2013/08/M/HS6/0004), with the dataset identifier ds003826 and version 3.0.0. The studies complied with the ethical standards outlined in the Declaration of Helsinki. This dataset is known for its open accessibility, public availability, and lack of usage restrictions. The study encompassed 123 participants, divided into two cohorts: 65 early chronotype (EC) and 58 late chronotype (LC) individuals. Each group was balanced in terms of gender, comprising 80 females and 43 males, aged between 18 and 35 years.

The dataset comprises structural T1-weighted magnetic resonance imaging (MRI) data from 123 young participants (80 females) aged between 18 and 35 years. It also includes assessments from questionnaires measuring the trait-like chronotype, sleep quality, and daytime sleepiness. Participants with self-reported psychiatric or neurological disorders and those currently taking medication were excluded from recruitment.

It has been proposed that the timing of MR image acquisition should be regulated because of the influence of experience-induced plasticity, which is linked to circadian brain changes and the impact of daily hormone fluctuations (such as cortisol and ACTH) [44,45]. Consequently, all brain scans were performed within the same timeframe each day, specifically between 5:20 p.m. and 8:55 p.m.

For each participant, circadian preference and the subjective amplitude of circadian rhythms were assessed using the Chronotype Questionnaire (ChQ). Daytime sleepiness and sleep quality were evaluated using the Epworth Sleepiness Scale (ESS) and the Pittsburgh Sleep Quality Index (PSQI), respectively. All questionnaire responses were collected before the brain imaging sessions. All participants were right-handed, possessed normal or corrected-to-normal vision, reported no neurological or psychiatric issues, and were drug-free. Additional inclusion criteria required participants to maintain a regular daily schedule without sleep debt, have no experience with shift work, and not have traveled across more than two time zones in the preceding two months.

MRI data were captured using a 3T scanner (Magnetom Skyra, Siemens) with either a 20-channel or 64-channel head/neck coil. High-resolution structural brain images were acquired using a T1 MPRAGE sequence, consisting of 176 sagittal slices, with a voxel size of 1 × 1 × 1.1 mm^3^, TR of 2300 ms, TE of 2.98 ms, flip angle of 9°, and GRAPPA acceleration factor of 2.

### 2.2. Pre-Processing Methods

**Figure 1:**
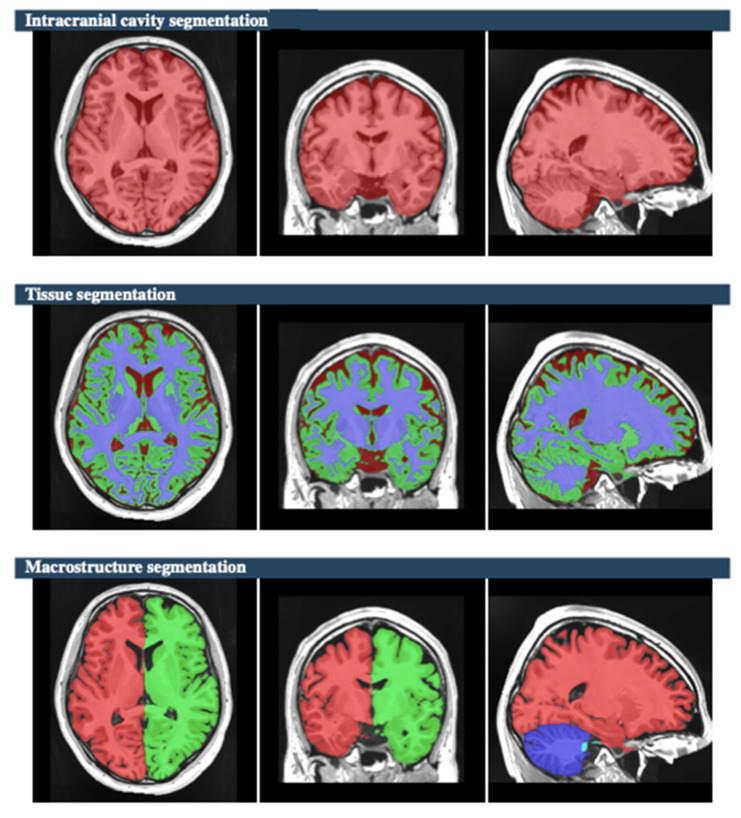
The process of non-local Intracranial Cavity Extraction (NICE), tissue classification, non-local hemisphere segmentation (NABS), and non-local subcortical structure segmentation [subject 10]. In the segmentation of the intracranial cavity, cerebral tissue is represented in red. For tissue segmentation, grey matter, white matter, and cerebrospinal fluid are indicated by light green, purple, and brown, respectively. In macrostructural segmentation, the right and left hemispheres are colored red and green, respectively, while the cerebellum is shown in blue.

The raw data of MR brain images were analyzed using volBrain Online software that is an automated and online MRI brain volumetry system (https://volbrain.net/, accessed on 1 December 2024). AssemblyNet, VolBrain and HIPS-monospectral pipelines were used to assess brain tissue, subcortical structures and hippocampal subfields, respectively. The high resolution T1-weighted imaging volumes underwent standard pre-processing steps: denoising, coarse inhomogenity correction, MNI space registration, fine inhomogenity correction and intensity normalization. Then, the segmentation process was applied, which consists of the following steps: spatially adaptive non-local means denoising, rough inhomogeneity correction, affining registration to MNI space, fine SPM-based inhomogeneity correction, intensity normalization, non-local Intracranial Cavity Extraction (NICE), tissue classification, non-local hemisphere segmentation (NABS) and non-local subcortical structure segmentation, as depicted in Figure 1.

### 2.3. Region of Interests (ROI) Selection

**Figure 2:**
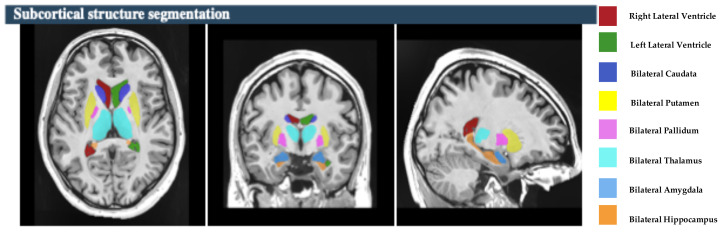
The process of subcortical structure segmentation. Right panel shows the color coded of hippocampal subfields that are overlaid onto the subject’s high-resolution T1-weighted anatomical scan [subject 10].

**Figure 3:**
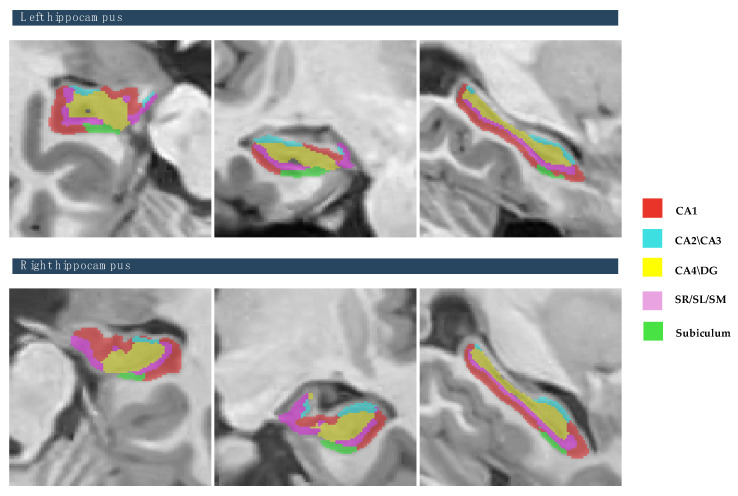
The process of hippocampal subfields segmentation based on the Winterburn atlas. Right panel shows the color coded of hippocampal subfields that are overlaid onto the subject’s high-resolution T1-weighted anatomical scan [subject 10].

The study focused on eight subcortical structures, divided into right and left hemispheres: the putamen, caudate, pallidum, thalamus, hippocampus, amygdala, and nucleus accumbens, and lateral ventricles, as depicted in Figure 2. Also, this study focused on the five hippocampal subfields, divided into right and left hemispheres: cornu ammonis 1 (CA1), cornu ammonis 2 and 3 (CA2–CA3), cornu ammonis 4 and the granule cell layer of dentate gyrus (CA4-DG), the strata radiatum/lacunosum/moleculare (SR-SL-SM), and Subiculum, as depicted in Figure 3. The volumetric measurements of the global tissue estimation (GM, WM, and CSF), macrostructures (cerebrum, cerebellum, vermis, and brainstem), subcortical structures (putamen, caudate, pallidum, thalamus, hippocampus, amygdala, nucleus accumbens, and lateral ventricles), hippocampal subfields (cornu ammonis 1 (CA1), cornu ammonis 2 and 3 (CA2–CA3), cornu ammonis 4, and the granule cell layer of dentate gyrus (CA4-DG), strataum radiatum/lacunosum/moleculare (SR-SL-SM) and Subiculum) were extracted.

### 2.4. ICV Normalization Methods

The volume of subcortical structures and hippocampal subfields were normalized by the total intracranial volumes (ICV) using the following equation:

Volume adjusted = Volume raw − β (ICV raw − ICV mean) (1)

where Volume adjusted is the normalized ROIs volumes, Volume raw is the absolute volume of ROIs that is extracted from the raw data, β is the slope of the regression line between ICV and the volume of the region of interests (ROIs), ICV raw is the intracranial volume of individuals that is extracted from the raw data, and ICV mean is the mean of the intracranial volume. All values are measured in cubic centimeters (cm^3^).

### 2.5. Hemispheric Asymmetries Measurements

An additional parameter is the asymmetry index (AI) that was applied, which is the difference between the volume of the right and left subcortical structures divided by their mean volume. This resulted in positive values (leftward asymmetry), negative values (rightward asymmetry), and zero values (non-directional asymmetry).

Asymmetry Index (AI) = (Volume L − Volume R)/(Volume L + Volume R) × 2(2)

where Volume L is the normalized ROIs volumes in the right hemisphere, and Volume R is the normalized ROIs volumes in the left hemisphere.

### 2.6. Statistical Analysis

All the statistical analyses were conducted with DATAtab Software Inc. (2024): Online Statistics Calculator. DATAtab e.U. Graz, Austria. URL https://datatab.net. Statistical analysis was performed using normalized volumes sourced from the volBrain program.

For all the statistical analyses, when *p* < 0.05, the test result was considered statistically significant. Then, the null hypothesis was rejected. The demographic characteristics and the volumes of subcortical structures and hippocampus between the EC group and the LC group were compared using the independent sample t-test. The effect size was assessed using Cohen’s d test. The collinearity between the demographic characteristics was performed using Pearson’s test to identify the linear relationship between variables. A comparative analysis of the structures’ volume was applied differently for the EC group and the LC group and the correlation between structures’ volume and participants’ demographics.

Multiple linear regression analysis was applied for the given brain volumetric measurements (GM, WM, CSF, intracranial cavity (IC), Brain (GM and WM), cerebrum, cerebellum, brainstem, subcortical structures, and hippocampal subfields volumes). For each model, these brain volumetric measurements were assigned as dependent variables and demographic characteristics (age, gender, study groups, CHQ-AM, PSQI, and ESS) as independent variables. Bonferroni correction for multiple comparisons was then applied to the tests by dividing *p* values by the number of tests, *p* = 0.05/6 = 0.008. All results were thresholded at *p*  <  0.008 with Bonferroni correction.

## 3. Results

### 3.1. Demographics

The demographic features of the participants in this study are presented in Table 1. Using the CHQ-ME to categorize participants into EC and LC groups revealed a significant difference (t(121) = −20.5, *p* < 0.001) between the EC (16.78 ± 2.72) and LC (26.74 ± 2.63) groups. However, there was no significant difference between the EC and LC groups regarding age (t(121) = 1.35, *p* = 0.17), gender (Z = −0.1, *p* = 0.93), CHQ-AM (t(121) = −0.96, *p* = 0.33), PSQI (t(121) = −0.85, *p* = 0.39), and ESS (t(121) = −0.74, *p* = 0.46).

The collinearity test revealed that there is a linear relationship between CHQ-AM and PSQI (r = 0.28, *p* = 0.002), and no linear relationships were found between CHQ-ME and CHQ-AM (r = 0.006, *p* = 0.5), CHQ-ME and PSQI (r = 0.08, *p* = 0.35), CHQ-ME and ESS (r = 0.12, *p* = 0.17), CHQ-AM and ESS (r = 0.02, *p* = 0.82), and between PSQI and ESS (r = 0.16, *p* = 0.07), as noted in Table 2.

### 3.2. Volumetric Measurements of Tissue and Macrostructure Segmentation

The volumetric analysis of tissue segmentation revealed no significant difference between the EC and LC groups in terms of GM (t(121) = 0.67, *p* = 0.5), WM (t(121) = 0.58, *p* = 0.56), CSF (t(121) = −0.46, *p* = 0.6), total brain volume (GM + WM) (t(121) = 0.65, *p* = 0.51), and IC (t(121) = 0.42, *p* = 0.67), as detailed in Table 3. Similarly, the macrostructure segmentation analysis showed no significant difference between the EC and LC groups for the cerebrum (t(121) = 0.49, *p* = 0.62), cerebellum (t(121) = 1.72, *p* = 0.08), vermis (t(121) = 0.2, *p* = 0.84), and brainstem (t(121) = 1.29, *p* = 0.19), as indicated in Table 4.

### 3.3. Volumetric Measurements of Subcortical Structures Segmentation

**Figure 4:**
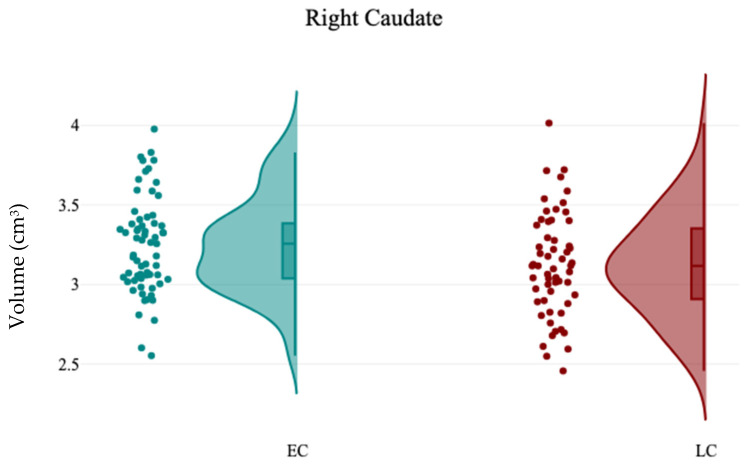
The raincloud chart shows the volumetric changes of right caudate between EC and LC groups.

**Figure 5:**
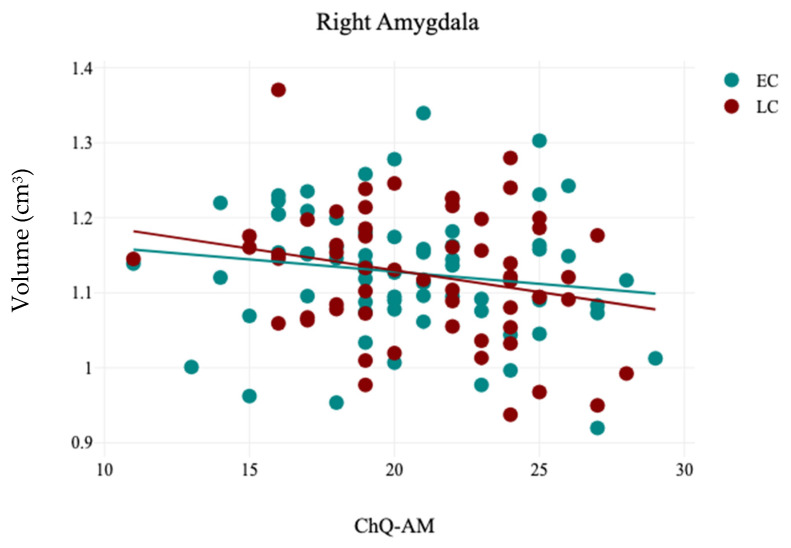
The results of the correlation analysis between CHQ-AM and the volume of right amygdala in EC and LC groups.

**Figure 6:**
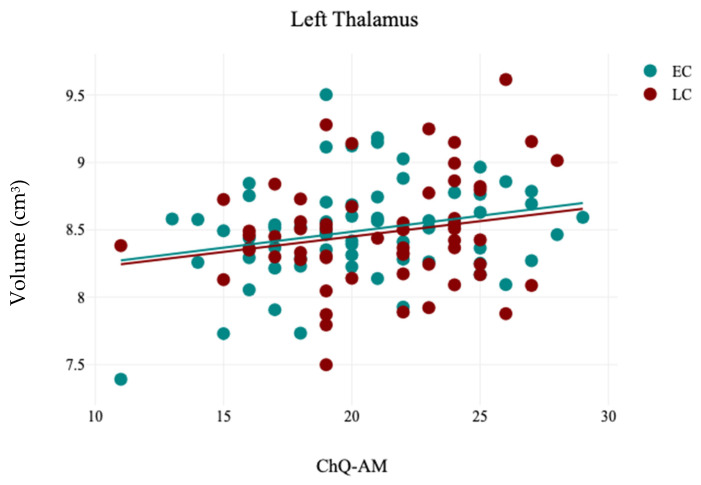
The results of the correlation analysis between CHQ-AM and the volume of left thalamus in EC and LC groups.

In contrast, the volumetric analysis of subcortical structures demonstrated a significant reduction in the volume of the right caudate for participants in the LC group (3.12 ± 0.32) compared to the EC group (3.24 ± 0.3), with t(121) = 2.13 and *p* = 0.03, as presented in Table 5 and Figure 4. No other significant differences were observed in the volumes of subcortical structures between the EC and LC groups. Additionally, the analysis of hemispheric laterality showed no significant difference in the Asymmetry Index (AI) between the EC and LC groups, as noted in Table 6. Correlation analysis between subcortical structures volumes and participants’ demographics revealed that the CHQ-AM significantly positively and negatively correlated with the left thalamus (r = 0.23, *p* = 0.01) and right amygdala volumes (r = −0.19, *p* = 0.03), as indicated in Table 7 and Figure 5 and Figure 6.

### 3.4. Volumetric Measurements of Hippocampal Subfields Segmentation

**Figure 7:**
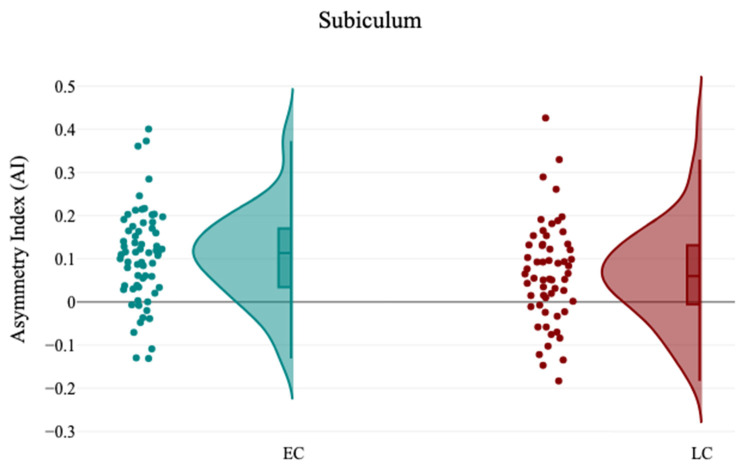
The raincloud chart shows the Asymmetry Index (AI) changes of Subiculum volume between EC and LC groups.

**Figure 8:**
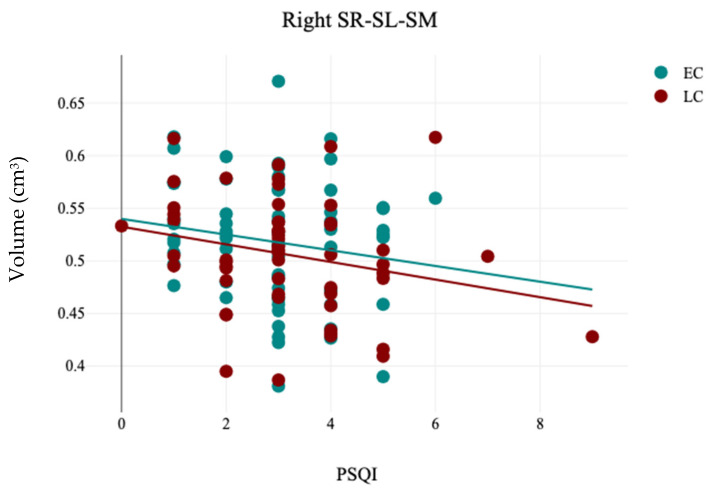
The results of the correlation analysis between PSQI and the volume of right hippocampal stratum (SR-SL-SM) in EC and LC groups.

**Figure 9:**
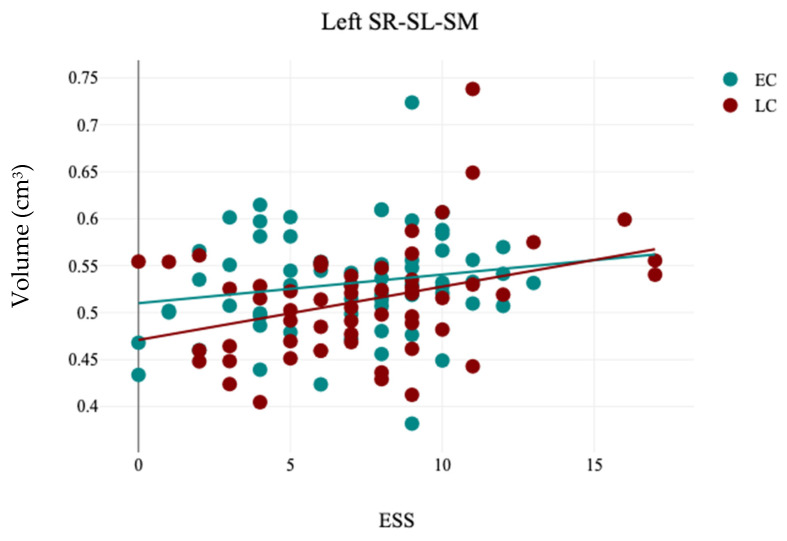
The results of the correlation analysis between ESS and the volume of left hippocampal stratum (SR-SL-SM) in EC and LC groups.

The volumetric analysis of hippocampal subfields revealed that there is no significant difference between the EC and LC groups regarding the volumes of hippocampal subfields (*p* > 0.05), as detailed in Table 8. In contrast, the analysis of hemispheric laterality showed a significant difference in the Asymmetry Index (AI) of the subiculum (t(121) = 2.0, *p* = 0.04) between the EC (0.11 ± 0.11) and LC (0.06 ± 0.12) groups, as noted in Table 9 and Figure 7. No other significant different was observed in the Asymmetry Indices of the hippocampal subfields between the EC and LC groups, as detailed in Table 9. Correlation analysis between the hippocampal subfields volumes and participants’ demographics revealed that sleep quality measured by PSQI is significantly negatively correlated with the volume of the right SR-SL-SM (r = −0.21, *p* = 0.02), and ESS significantly positively correlated with the left SR-SL-SM (r = −0.24, *p* = 0.007), as noted in Table 10 and Figure 8 and Figure 9.

### 3.5. Regression Models

Multiple regression analysis developed 33 regression models that assessed the association between each volumetric measurement and demographic characteristics, as detailed in Table 11. In the analysis of tissue classification and macrostructures, gender was the only factor that was related to the volumes of gray matter (GM) (β = −0.63, *p* < 0.001), gray matter (GM) (β = −0.56, *p* < 0.001), cerebrospinal fluid (CSF) (β = −0.4, *p* < 0.001), the overall brain (GM + WM) (β = −0.62, *p* < 0.001), the intracranial cavity (IC) (β = −0.64, *p* < 0.001), cerebrum (β = −0.61, *p* < 0.001), cerebellum (β = −0.45, *p* < 0.001), and vermis (β = −0.48, *p* < 0.001). No associations were found between the demographic characteristics factors (age, gender, study group, CHQ-AM, PSQI, and ESS) and the volumetric measurements at the level of subcortical structures. Regarding hippocampal subfields, the volumes of the left subiculum and SR-SL-SM were found to be associated with age (β = 0.27, *p* = 0.003) and ESS (β = 0.28, *p* = 0.002), respectively.

## 4. Discussion

In this study, the association between sleep chronotype and brain morphometry has been investigated using four self-reported questionnaire assessments (CHQ-ME, CHQ-AM, PSQI, and ESS) and brain volumetric analysis at four levels (tissue classification, macrostructures, subcortical structures, and hippocampal subfields). Three different analyses were applied (independent sample *t*-test, Pearson’s correlation analysis, and Multiple linear regression) to assess the association between sleep chronotype and brain morphometry. Various subcortical structures and hippocampal subfields were found to be linked to chronotype. The LC group exhibited notable reductions in the right caudate and the left SR-SL-SM when compared to the EC group. Additionally, a significant leftward hemispheric laterality of the subiculum was observed in the EC group relative to the LC group. Furthermore, the CHQ-AM demonstrated significant positive and negative correlations with the volumes of the left thalamus and right amygdala, respectively. The PSQI showed a significant negative correlation with the right SR-SL-SM, while the ESS indicated a significant positive correlation with the left SR-SL-SM. Multiple regression analysis revealed that changes in chronotype preferences are associated with variations in the volumetric measurements of the left SR-SL-SM region.

The caudate nucleus, located deep within the brain near the thalamus, is essential for various higher neurological functions, including movement planning, learning, memory, reward, motivation, emotion, and romantic interactions [46]. Research has indicated that the caudate is a crucial component in the neuronal network linked to insomnia [47]. In the current study, it is observed that the volume of the right caudate was significantly reduced in the LC group compared to the EC group. Consistent with this finding, previous studies have shown that sleep duration correlates with caudate volume in both younger [48] and older adults [49]. These results suggest that the caudate nuclei play a vital role in sleep, potentially due to their involvement in reward and sensory processing, as well as the regulation of cortical excitability [47].

The amygdala, situated beneath the cortex and part of the limbic system, plays a key role in managing emotions, motivation, and memory. Growing evidence suggests that it is essential for the consolidation of emotional memories during sleep [50]. In this study, a negative correlation was found between the volume of the right amygdala and the CHQ-AM. This aligns with findings from Stanford et al. (2022), who noted that the amygdala is a vital mediator of emotion and sleep [51]. Additionally, it was reported that poorer sleep quality was associated with increased functional connectivity between the right amygdala and the postcentral gyrus, while also showing decreased connectivity between the amygdala and the posterior cerebellar lobe [52]. Gong et al. (2019) also found a link between insomnia severity and the centromedial right amygdala [53]. Furthermore, a later chronotype was significantly related to reduced functional connectivity between the amygdala and the dorsal anterior cingulate cortex [54]. These results suggest that the human amygdala is influenced by the same global sleep–wake factors as other brain regions [55], supporting the theory of impaired emotion regulation in individuals with a late chronotype [54].

The current study revealed a significant positive correlation between the volume of the left thalamus and the CHQ-AM. Supporting this finding, another study has indicated that alterations in the regional shape of the left thalamus are associated with a late chronotype in young adults, which suggests that a late chronotype could be a potential risk factor for sleep-related behavioral and mental challenges in this demographic [56]. The thalamus significantly influences sleep, circadian rhythms, and melatonin production, as it initiates non-rapid eye movement sleep and is aided by melatonin in promoting sleep spindle formation [57].

The hippocampus, situated deep within the temporal lobe and part of the brain’s limbic system, plays a vital role in learning, emotions, and memory. Its importance in the context of neuropsychiatric disorders emphasizes its relevance for diagnosis and treatment evaluation [58]. However, the relationship between sleep quality and the volume loss patterns in the hippocampal subfields remains poorly understood [59]. Research has indicated that sleep deprivation affects hippocampal function [60,61]. Alperin (2021) discovered a link between poor sleep quality and reductions in volume in the left CA1, DG, and subiculum among cognitively healthy older adults [62]. Additionally, Yang et al. (2022) explored the volume change patterns in neurofunctional hippocampal subfields among insomnia patients, finding that atrophy in certain neurofunctional subfields was not only associated with insomnia but also posed a significant risk factor for its development [63]. In this study, while no significant correlation was found between total hippocampal volume and chronotype, specific subfields—namely the SR-SL-SM and subiculum—were connected to chronotypes. The hippocampal stratum (SR-SL-SM) features numerous apical dendrites and axonal fasciculi that are aligned with the internal surface of the CA, serving as critical gateways for two primary glutamatergic excitatory inputs directed to CA1 from CA3 and the entorhinal cortex [64]. Su et al. (2018) noted a correlation between the volume of the hippocampal stratum (SR-SL-SM) and clinical, as well as cognitive, assessments of disease severity [65]. Conversely, the subiculum is a significant target for CA1 projections from the hippocampus and functions as the main output pathway of the hippocampal formation [66]. De Looze et al. (2022) found a connection between sleep duration and subiculum volume [67]. These results indicate that individuals with poor sleep quality and duration may be at an increased risk for atrophy in the hippocampal subfields.

Hemispheric asymmetries are crucial in various cognitive functions, including memory [68], language [69], spatial attention [70], and emotion [71]. In this study, a notable leftward hemispheric laterality of the subiculum was found in the EC group compared to the LC group. Joo et al. (2014) noted that the asymmetrical hemispheric volume of the dentate gyrus (DG) and CA3-4 was linked to deficits in verbal domain functions among patients suffering from chronic primary insomnia [38]. These findings may shed light on the pathophysiological mechanisms that render individuals with sleep disturbances susceptible to cognitive decline.

This study offers important insights into the relationship between brain morphometry and chronotype; however, it is crucial to acknowledge several limitations that could influence the interpretation of the results. A major limitation is the small sample size of just 123 participants, which may limit the generalizability of the findings. Additionally, the study’s dependence on self-reported data presents another limitation, as participants might not accurately reflect their sleep experiences, leading to potential biases. Future research should aim for a larger and more diverse population and utilize a longitudinal design to gain a deeper understanding of the relationship between brain morphometry and chronotype.

## 5. Conclusions

This research identifies a relationship between chronotypes and the volumes of subcortical structures, including specific regions of the hippocampus. Notably, variations in chronotype are linked to the volumes of the caudate, thalamus, amygdala, hippocampal stratum, and subiculum. The findings enhance our understanding of the role of sleep chronotypes in the neural mechanisms of these brain areas. Additionally, brain volumetric analysis serves as a useful method for examining the connection between brain morphology and chronotype, with potential relevance for other neurological conditions.

## Figures and Tables

**Table 1:** The demographic characteristics of the participants involved in this study.

Variables	Groups	Desciptive Analysis					Independent Samples *t*-Test		
		N	Min	Max	Mean ± Std.	95% CI for Mean	t	*p*	*Cohen’s d*
Age	EC	65	19	35	24.75 ± 3.73	23.83–25.68	1.35	0.179	0.24
	LC	58	18	35	23.86 ± 3.56	22.93–24.8			
ChQ-ME	EC	65	11	21	16.78 ± 2.72	16.11–17.46	−20.5	<0.001	3.72
	LC	58	22	32	26.74 ± 2.63	26.05–27.43			
ChQ-AM	EC	65	11	29	20.35 ± 3.94	19.38–21.33	−0.96	0.336	0.17
	LC	58	11	28	21.02 ± 3.65	20.06–21.98			
PSQI	EC	65	1	6	2.95 ± 1.27	2.64–3.27	−0.85	0.395	0.15
	LC	58	0	9	3.17 ± 1.57	2.76–3.59			
ESS	EC	65	0	13	6.89 ± 3.2	6.1–7.69	−0.74	0.464	0.13
	LC	58	0	17	7.34 ± 3.63	6.39–8.3			

**Table 2:** The results of collinearity test for self-assessment of Chronotype questionnaires.

	ChQ-ME	ChQ-AM	PSQI	ESS
ChQ-ME	1	0.06	0.08	0.12
ChQ-AM	0.06	1	0.28	0.02
PSQI	0.08	0.28	1	0.16
ESS	0.12	0.02	0.16	1

**Table 3:** The volumetric results of tissue segmentation analysis between EC and LC groups.

Volume (cm^3^)	Group	Desciptive Analysis		Independent Samples *t*-Test		
		Mean ± Std.	95% CI for Mean	t	*p*	*Cohen’s d*
Grey Matter (GM)	EC	804.01 ± 61.09	788.87–819.15	0.67	0.50	0.12
	LC	796.55 ± 63.06	779.96–813.14			
White Matter (WM)	EC	479.33 ± 44.37	468.33–490.32	0.58	0.56	0.11
	LC	474.68 ± 43.99	463.11–486.25			
Cerebro Spinal Fluid (CSF)	EC	155.89 ± 36.62	146.81–164.97	−0.46	0.64	0.08
	LC	158.97 ± 38.03	148.97–168.97			
Brain (GM + WM)	EC	1283.34 ± 101.94	1258.08–1308.6	0.65	0.51	0.12
	LC	1271.23 ± 104.68	1243.7–1298.77			
Intracranial Cavity (IC)	EC	1457.73 ± 124.13	1426.97–1488.49	0.42	0.67	0.08
	LC	1448.26 ± 123.7	1415.72–1480.79			

**Table 4:** The volumetric results of macrostructure segmentation analysis between EC and LC groups.

Volume (cm^3^)	Group	Desciptive Analysis		Independent Samples *t*-Test		
		Mean ± Std.	95% CI for Mean	t	*p*	*Cohen’s d*
Cerebrum	EC	1131.53 ± 93.03	1108.47–1154.58	0.49	0.62	0.09
	LC	1123.19 ± 96.8	1097.73–1148.65			
Cerebellum	EC	139.29 ± 12.41	136.22–142.37	1.72	0.08	0.31
	LC	135.58 ± 11.4	132.58–138.57			
Vermis	EC	12.52 ± 1.51	12.14–12.89	0.2	0.84	0.04
	LC	12.47 ± 1.28	12.13–12.81			
Brainstem	EC	18.5 ± 1.98	18.01–18.99	1.29	0.19	0.23
	LC	18.05 ± 1.85	17.57–18.54			

**Table 5:** The volumetric results of subcortical structures analysis between EC and LC groups.

Volume (cm^3^)	Group	Right Hemisphere Independent Samples *t*-Test					Left Hemisphere Independent Samples *t*-Test				
		Mean ± Std.	95% CI for Mean	t	*p*	*Cohen’s d*	Mean ± Std.	95% CI for Mean	t	*p*	*Cohen’s d*
Accumbens	EC	0.48 ± 0.06	0.46–0.49	0.22	0.82	0.04	0.54 ± 0.06	0.52–0.55	0.04	0.96	0.01
	LC	0.48 ± 0.07	0.46–0.49				0.54 ± 0.07	0.52–0.56			
Amygdala	EC	1.13 ± 0.08	1.1–1.15	0.2	0.84	0.04	1.07 ± 0.08	1.05–1.09	−0.9	0.37	0.16
	LC	1.12 ± 0.09	1.1–1.15				1.09 ± 0.08	1.07–1.11			
Caudate	EC	3.24 ± 0.3	3.17–3.32	2.13	0.03	0.38	3.19 ± 0.29	3.11–3.26	1.78	0.07	0.32
	LC	3.12 ± 0.32	3.04–3.21				3.09 ± 0.33	3.0–3.17			
Hippocampus	EC	3.83 ± 0.27	3.76–3.9	1.6	0.11	0.29	3.76 ± 0.27	3.69–3.83	1.16	0.25	0.21
	LC	3.75 ± 0.23	3.69–3.81				3.7 ± 0.26	3.63–3.77			
Pallidum	EC	1.67 ± 0.12	1.64–1.7	0.5	0.61	0.09	1.78 ± 0.11	1.75–1.81	0.94	0.35	0.17
	LC	1.66 ± 0.12	1.63–1.69				1.76 ± 0.12	1.73–1.79			
Putamen	EC	4.82 ± 0.34	4.73–4.9	−0.33	0.74	0.06	4.82 ± 0.34	4.74–4.91	−0.77	0.42	0.14
	LC	4.84 ± 0.38	4.74–4.94				4.88 ± 0.4	4.77–4.98			
Thalamus	EC	8.53 ± 0.38	8.44–8.62	−0.6	0.55	0.11	8.49 ± 0.36	8.4–8.58	0.3	0.76	0.05
	LC	8.57 ± 0.41	8.47–8.68				8.47 ± 0.4	8.37–8.58			
Lateral ventricle	EC	6.72 ± 3.03	5.97–7.47	−0.36	0.71	0.07	7.03 ± 2.87	6.32–7.74	−0.66	0.51	0.12
	LC	6.94 ± 3.73	5.96–7.93				7.43 ± 3.74	6.44–8.41			

**Table 6:** The Asymmetry Index (AI) results of subcortical structures analysis between EC and LC groups.

Asymmetry Index (AI)	Group	Desciptive Analysis		Independent Samples *t*-Test		
		Mean ± Std.	95% CI for Mean	t	*p*	*Cohen’s d*
Accumbens volume	EC	0.12 ± 0.09	0.1–0.14	−0.4	0.69	0.07
	LC	0.13 ± 0.09	0.1–0.15			
Amygdala volume	EC	−0.05 ± 0.06	−0.06–−0.03	−1.41	0.16	0.25
	LC	−0.03 ± 0.06	−0.05–−0.02			
Caudate volume	EC	−0.02 ± 0.03	−0.03–−0.01	−0.94	0.35	0.17
	LC	−0.01 ± 0.03	−0.02–0			
Hippocampus volume	EC	−0.02 ± 0.04	−0.03–−0.01	−0.52	0.6	0.09
	LC	−0.01 ± 0.04	−0.03–0			
Pallidum volume	EC	0.07 ± 0.03	0.06–0.07	0.86	0.38	0.16
	LC	0.06 ± 0.03	0.05–0.07			
Putamen volume	EC	0 ± 0.02	0–0.01	−1.71	0.08	0.31
	LC	0.01 ± 0.02	0–0.01			
Thalamus volume	EC	0 ± 0.02	−0.01–0	1.91	0.059	0.34
	LC	−0.01 ± 0.02	−0.02–−0.01			
Lateral ventricle volume	EC	0.06 ± 0.33	−0.02–0.14	−0.28	0.77	0.05
	LC	0.08 ± 0.22	0.02–0.13			

**Table 7:** The results of the correlation analysis between the scores from self-reported chronotype questionnaires and the volumes of subcortical structures.

Volume	Hemisphere	Demographics			
		CHQ-ME	CHQ-AM	PSQI	ESS
Accumbens	R	0.01	0.03	−0.01	0.08
	L	−0.01	0.05	0.03	0.09
Amygdala	R	0.02	−0.19	−0.18	0
	L	0.11	−0.14	−0.13	0.01
Caudate	R	−0.1	0.06	0	0.12
	L	−0.06	0.06	−0.05	0.13
Hippocampus	R	−0.13	−0.02	−0.05	0.04
	L	−0.1	0.03	0.03	0.1
Pallidum	R	−0.09	0.09	−0.02	−0.07
	L	−0.09	0.01	−0.09	−0.05
Putamen	R	0.05	0.07	−0.1	0.02
	L	0.1	0.12	−0.05	0.02
Thalamus	R	0.08	0.14	0.03	0.07
	L	0.01	0.23	0.13	0.1
Lateral ventricle	R	0.01	−0.03	0.06	−0.06
	L	0.07	0.02	0.06	0.04

**Table 8:** The volumetric results of hippocampal subfields analysis between EC and LC groups.

Volume (cm^3^)	Group	Right Hemisphere		Independent Samples *t*-Test			Left Hemisphere		Independent Samples *t*-Test		
		Mean ± Std.	95% CI for Mean	t	*p*	*Cohen’s d*	Mean ± Std.	95% CI for Mean	t	*p*	*Cohen’s d*
CA1	EC	0.92 ± 0.11	0.89–0.95	1.15	0.25	0.21	0.17 ± 0.03	0.16–0.18	0.72	0.47	0.13
	LC	0.9 ± 0.1	0.87–0.93				0.17 ± 0.03	0.16–0.18			
CA2-CA3	EC	0.21 ± 0.04	0.2–0.21	1.81	0.07	0.33	0.9 ± 0.1	0.87–0.92	0.67	0.5	0.12
	LC	0.19 ± 0.03	0.19–0.2				0.89 ± 0.1	0.86–0.91			
CA4-DG	EC	0.73 ± 0.07	0.72–0.75	0.19	0.84	0.03	0.71 ± 0.08	0.69–0.73	0.14	0.88	0.03
	LC	0.73 ± 0.09	0.71–0.75				0.71 ± 0.1	0.68–0.74			
SR-SL-SM	EC	0.52 ± 0.06	0.5–0.53	1.19	0.23	0.21	0.53 ± 0.06	0.52–0.54	1.8	0.07	0.32
	LC	0.51 ± 0.05	0.49–0.52				0.51 ± 0.06	0.5–0.53			
Subiculum	EC	0.27 ± 0.04	0.26–0.28	−0.49	0.62	0.09	0.3 ± 0.05	0.29–0.31	1.04	0.3	0.19
	LC	0.28 ± 0.04	0.27–0.29				0.29 ± 0.04	0.28–0.3			

**Table 9:** The Asymmetry Index (AI) results of hippocampal subfields analysis between EC and LC groups.

Asymmetry Index (AI)	Group	Desciptive Analysis		Independent Samples *t*-Test		
		Mean ± Std.	95% CI	*T*	*p*	*Cohen’s d*
CA1	EC	−0.02 ± 0.08	−0.04–0	−0.68	0.49	0.12
	LC	−0.01 ± 0.08	−0.03–0.01			
CA2-CA3	EC	−0.18 ± 0.17	−0.22–−0.13	−0.93	0.35	0.17
	LC	−0.15 ± 0.16	−0.19–−0.11			
CA4-DG	EC	−0.03 ± 0.09	−0.05–−0.01	−0.02	0.98	0
	LC	−0.03 ± 0.09	−0.06–−0.01			
SR-SL-SM	EC	0.03 ± 0.09	0–0.05	0.79	0.42	0.14
	LC	0.01 ± 0.09	−0.01–0.04			
Subiculum	EC	0.11 ± 0.11	0.08–0.13	2	0.04	0.36
	LC	0.06 ± 0.12	0.03–0.1			

**Table 10:** The results of the correlation analysis between the scores from self-reported chronotype questionnaires and the volumes of hippocampal subfields.

	Hemisphere	Demographics			
		CHQ-ME	CHQ-AM	PSQI	ESS
CA1	RH	−0.09	−0.05	−0.08	0.02
	LH	−0.06	−0.03	0.02	0.1
CA2-CA3	RH	−0.15	0.07	−0.04	0.12
	LH	−0.02	−0.02	0.07	0.14
CA4-DG	RH	−0.01	−0.1	−0.13	0.08
	LH	0	0	0.01	0.09
SR-SL-SM	RH	−0.1	−0.03	−0.21	0.13
	LH	−0.1	−0.05	−0.03	0.25
Subiculum	RH	−0.02	0.03	−0.03	−0.03
	LH	−0.16	0.09	0.02	−0.08

**Table 11:** Association between brain volumetric measurements (dependent variable) and demographic characteristics (independent variables).

	Age		Gender		Study Group		CHQ-AM		PSQI		ESS	
	β	*p*	β	*p*	β	*p*	β	*p*	β	*p*	β	*p*
Tissue classification												
Grey Matter (GM)	−0.08	0.29	−0.63	<0.001	−0.35	0.02	0.15	0.04	−0.03	0.71	0.04	0.57
White Matter (WM)	0.08	0.30	−0.56	<0.001	−0.26	0.11	0.18	0.03	0.01	0.9	0.05	0.52
Cerebro Spinal Fluid (CSF)	0.19	0.02	−0.4	<0.001	−0.29	0.1	0.04	0.67	0.05	0.57	−0.03	0.75
Brain (GM + WM)	−0.01	0.878	−0.62	<0.001	−0.32	0.04	0.17	0.034	−0.01	0.874	0.05	0.539
Intracranial Cavity (IC)	0.05	0.501	−0.64	<0.001	−0.36	0.02	0.15	0.046	0	0.958	0.03	0.672
Macrostructures												
Cerebrum	0.01	0.924	−0.61	<0.001	−0.31	0.055	0.16	0.049	−0.01	0.889	0.05	0.499
Cerebellum	−0.15	0.07	−0.45	<0.001	−0.27	0.126	0.18	0.035	−0.02	0.792	0	0.99
Vermis	−0.05	0.557	−0.48	<0.001	−0.26	0.136	0.16	0.065	0.02	0.776	−0.06	0.478
Brainstem	0.2	0.034	0.03	0.709	0.06	0.762	0.12	0.227	−0.06	0.499	0.03	0.759
Subcortical structures												
RH Accumbens	−0.11	0.23	0	1	−0.12	0.538	0.03	0.793	−0.03	0.747	0.07	0.445
LH Accumbens	−0.08	0.379	−0.09	0.322	0.01	0.952	0.05	0.605	0	0.968	0.1	0.275
RH Amygdala	0.01	0.879	−0.09	0.358	−0.15	0.435	−0.14	0.149	−0.13	0.164	0.02	0.798
LH Amygdala	−0.01	0.902	−0.11	0.256	−0.07	0.713	−0.1	0.31	−0.11	0.256	0.03	0.75
RH Caudate	−0.04	0.684	0.12	0.205	−0.43	0.027	0.06	0.495	−0.03	0.727	0.1	0.277
LH Caudate	−0.03	0.744	0.13	0.173	−0.44	0.022	0.08	0.404	−0.09	0.316	0.12	0.201
RH Hippocampus	0.13	0.179	−0.13	0.165	−0.17	0.376	0.04	0.671	−0.04	0.671	0.07	0.437
LH Hippocampus	0.21	0.021	−0.12	0.181	−0.11	0.567	0.08	0.387	0.01	0.889	0.13	0.152
RH Pallidum	−0.02	0.814	−0.12	0.224	−0.09	0.656	0.12	0.206	−0.02	0.838	−0.03	0.718
LH Pallidum	−0.05	0.587	−0.14	0.141	−0.19	0.332	0.12	0.221	−0.08	0.425	−0.03	0.769
RH Putamen	−0.07	0.456	−0.15	0.105	−0.07	0.717	0.15	0.13	−0.12	0.208	0.05	0.567
LH Putamen	−0.03	0.742	−0.16	0.098	−0.06	0.763	0.19	0.054	−0.11	0.234	0.07	0.469
RH Thalamus	0.12	0.194	−0.01	0.921	−0.11	0.593	0.16	0.098	−0.02	0.801	0.06	0.539
LH Thalamus	0.18	0.055	−0.03	0.702	−0.22	0.257	0.25	0.01	0.06	0.533	0.08	0.363
RH Lateral ventricle	0.23	0.014	0.02	0.791	−0.19	0.316	0.06	0.524	0.02	0.844	−0.07	0.453
LH Lateral ventricle	0.13	0.152	0.09	0.332	−0.19	0.339	0.05	0.619	−0.03	0.722	−0.02	0.863
Hippocapmal subfields												
RH CA1	0.18	0.061	−0.03	0.74	−0.14	0.484	0	0.97	−0.07	0.451	0.05	0.592
LH CA1	0.15	0.112	−0.1	0.281	−0.05	0.801	0	0.964	0.02	0.852	0.12	0.193
RH CA2-CA3	0.17	0.069	0.02	0.851	−0.15	0.429	0.12	0.207	−0.08	0.412	0.14	0.127
LH CA2-CA3	0.18	0.057	0.02	0.805	−0.18	0.352	−0.01	0.956	0.06	0.564	0.13	0.174
RH CA4-DG	−0.07	0.45	0	0.999	0.01	0.957	−0.08	0.442	−0.12	0.208	0.1	0.311
LH CA4-DG	0	0.968	0.05	0.62	−0.03	0.868	0	0.971	0	0.974	0.08	0.407
RH SR-SL-SM	0.08	0.354	−0.09	0.338	−0.08	0.668	0.07	0.47	−0.24	0.013	0.19	0.039
LH SR-SL-SM	0.08	0.379	−0.1	0.272	−0.28	0.137	0	0.992	−0.05	0.603	0.28	0.002
RH Subiculum	0.22	0.021	0.04	0.688	0.25	0.203	0.06	0.567	−0.04	0.675	−0.01	0.939
LH Subiculum	0.27	0.003	0.04	0.663	0.17	0.366	0.12	0.193	0.01	0.929	−0.05	0.581

## Data Availability

The original contributions presented in this study are included in the article. Further inquiries can be directed to the corresponding author.

## Abbreviations

The following abbreviations are used in this manuscript:

ChQ-ME	Morningness–Eveningness scale of the Chronotype Questionnaire
ChQ-AM	Amplitude Scale of Chronotype Questionnaire
ESS	Epworth Sleepiness Scale
PSQI	Pittsburgh Sleep Quality Index
EC	Early chronotype
LC	Late chronotype
NICE	Non-local Intracranial Cavity Extraction
NABS	Non-local hemisphere segmentation
CA	Cornu ammonis
DG	Dentate Gyrus
SR-SL-SM	Strata radiatum/lacunosum/moleculare
ICV	Intracranial Volumes
AI	Asymmetry Index
GM	Grey Matter
WM	White Matter
CSF	Cerebro Spinal Fluid
IC	Intracranial Cavity
